# Microbiota shape the colon epithelium controlling inter-crypt absorptive goblet cells via butyrate–GP R109A signalling

**DOI:** 10.1080/19490976.2025.2573045

**Published:** 2025-11-09

**Authors:** Vinícius Dias Nirello, Nathália Araújo, Helder Carvalho de Assis, Mar Moreno-Gonzalez, Paula Ruiz, Pollyana Ribeiro Castro, Nicolas G. Shealy, Catherine Shelton, Mariane Font Fernandes, Sarah de Oliveira, Mariana Boroni, Bernhard Ryffel, Mariana Xavier Byndloss, Naiara Beraza, Marco Aurélio Ramirez Vinolo, Patrick Varga-Weisz

**Affiliations:** a Department of Genetics, Evolution, Microbiology, and Immunology, Institute of Biology, University of Campinas, Campinas, Brazil; b Food, Microbiome and Health Institute Strategic Programme, Quadram Institute Bioscience, Norwich Research Park, Norwich, UK; c Department of Pathology, Microbiology and Immunology, Vanderbilt University Medical Center, Nashville, TN, USA; d Bioinformatics and Computational Biology Lab, Brazilian National Cancer Institute (INCA), Rio de Janeiro, Brazil; e Laboratory of Immuno-Neuro Modulation, INEM, UMR7355 CNRS and University of Orleans, Orleans, France; f Howard Hughes Medical Institute, Vanderbilt University Medical Center, Nashville, TN, USA; g Experimental Medicine Research Cluster (EMRC), Campinas, Brazil; h National Institute of Science and Technology in Modeling Human Complex Diseases with 3D Platforms (INCT Model3D), São Paulo, Brazil; i Obesity and Comorbidities Research Center (OCRC), University of Campinas, Campinas, Brazil; j School of Life Sciences, University of Essex, Colchester, UK

**Keywords:** Single-cell transcriptomics, colon epithelium, butyrate, GPR109A, HCAR2, aging

## Abstract

The colonic epithelium is a key interface between the gut microbiota and the host. How microbiota-derived signals influence epithelial cell identity and function remains incompletely understood. Here, we used single-cell transcriptomics, antibiotic-mediated microbiota depletion, germ-free mice and colonization experiments in mice to uncover cell-type-specific responses to microbiota changes, highlighting changes in the cell composition and functional diversities in enterocytes. Our analysis demonstrates that the microbiota control the absorptive profile of the colon epithelial cells and reveals non-canonical inter-crypt goblet cells as microbiota-responsive constituents that combine absorptive and secretory features and whose abundance is regulated by the gut microbiota. We found that their number is suppressed through the short-chain fatty acid butyrate and its receptor GPR109A. Analysis in mouse and humans indicates that the expansion of this hybrid population increases with age and that this expansion is driven by microbiome changes. Our work reveals a previously unrecognized level of epithelial plasticity driven by microbial triggers and highlights butyrate, acting as a signaling molecule that shapes the colon micro-anatomy.

## Graphical abstract

**Figure:**
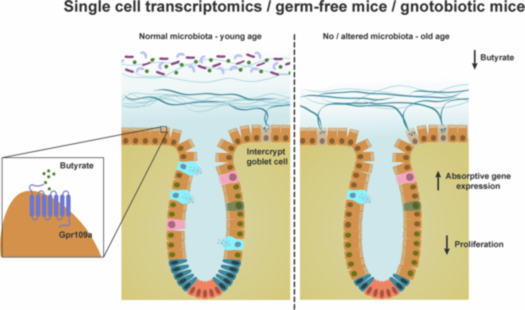

## Introduction

Owing to its dual role in nutrient absorption and protection against external threats, the intestinal tract relies on specialized intestinal epithelial cells (IECs) that form a barrier between the host and the gut content, including the microbiota. Various types of mature cells perform specialized functions in the intestine, such as nutrient absorption, mucus secretion, metabolic control, and immune regulation. The self-renewal of the intestinal epithelium, which is necessary due to the persistent aggression by luminal contents, is driven by a small population of multipotent intestinal stem cells (ISCs) residing in specialized niches within the crypts, invaginations that structure the intestinal epithelium.^1^ These ISCs generate highly proliferative and short-lived progenitors called transient amplifying (TA) cells, which differentiate into absorptive (enterocytes) or secretory (goblet cells, enteroendocrine cells) cell lineages while migrating toward the top of the crypt.^2^

Enterocytes in the colon, also referred to as colonocytes, are the predominant cell type in the intestinal epithelium. They play a pivotal role in absorbing nutrients such as minerals, short-chain fatty acids, vitamins, and water while also actively communicating with the immune system. Enterocytes serve as a barrier against microbial invasion and produce antimicrobial peptides (AMPs).^3^^,^^4^ Goblet cells, meanwhile, produce the mucus layer that further restricts microbial interactions with underlying tissues by expressing mucin-2 (MUC2), Fcγ binding protein (FCGBP), and calcium-activated chloride channel regulator 1 (CLCA1).^5–8^

The composition of cells in the intestine exhibits substantial differences across regions, demonstrating the complexity of epithelial subtypes. Moreover, the same cell types organize into distinct neighborhoods and communities, highlighting the diverse functional niches present in the intestine.^9^ For instance, enterocyte function in the small intestine is zonated along the villus axis, with enterocytes traversing various cell states during migration along the villus.^10^ Immune-modulatory programs are also zonated, and secretory cells exhibit heterogeneous positions within the epithelium. There is elevated expression of immune-modulatory genes in villus tip-resident goblet and tuft cells, along with heterogeneous localizations of enteroendocrine cells.^11^ Multiple distinct goblet cell subsets further contribute to this complexity.^12^

Modulations in the microbiota and microbiota-derived metabolites drive plasticity in the IEC composition.^13^ The microbial conversion of dietary fiber in the gut leads to the synthesis of three major short-chain fatty acids (SCFAs): acetate, propionate, and butyrate, which have profound effect on intestinal physiology. Butyrate serves as the preferred energy source for colonocytes and is primarily consumed locally, while other absorbed SCFAs drain into the portal vein.^14^ SCFAs can affect IECs through receptors such as GPR43 (FFAR2), which is linked to acetate and propionate, and GPR109A (HCAR2), which is activated by niacin, *β*-D-hydroxybutyrate (BHB), and butyrate.^15^ Recent studies have described how propionate produced by *Bacteroides* promotes goblet cell differentiation through the propionate receptor GPR41 (FFAR3),^16^ whereas butyrate restricts tuft cell differentiation via the internalisation and inhibition of histone deacetylase HDAC3.^17^

Antibiotic usage can have pervasive and long-lasting effects on the composition and functionality of the gut microbiota, potentially affecting the overall health and immune response of the host.^18–20^ Germ-free (GF) mice exhibit metabolic changes such as alterations in bile acid pools, incretin release, and glucose homeostasis.^21^ Furthermore, different diets can modulate microbiota composition and alter microbiota-derived metabolite levels. We have shown that an inulin-rich diet significantly affects colon and caecum anatomy both at the gross and cellular levels by altering the composition of the microbiota.^22^ Recent efforts have focused on understanding the specific impacts of microbiota composition in the gut, with a specific emphasis on the small intestine using GF and specific pathogen-free (SPF) models.^23^ However, considering that the highest density of the microbiota is in the colon and that this tissue is strongly focused on microbiota contention, it is essential to understand how microbiota modulation affects IECs specialization in the colon.

In this new study, we employ different modulations of the microbiota and its derivatives to understand how the gut microbiota shapes the intestinal epithelium. Our analysis uncovers cell-type-specific responses to microbiota changes and highlights the functional diversities in enterocytes. We identify inter-crypt goblet cells that combine absorptive and secretory features as highly microbiota-responsive cells and elucidated the mechanisms by which these cells are regulated by the microbiota. Our work illustrates how the microbiota shapes colon epithelium cellular anatomy and function.

## Materials and methods

### Mice

Adult C57BL6/J mice were obtained from the Multidisciplinary Centre for Biological Investigation at the University of Campinas (CEMIB). Whole-body GPR43 KO mice were obtained from CEMIB, originally generated by Charles Mackay (Deltagen).^24^ All animals were housed in the animal facility at the Department of Genetics, Evolution, Microbiology, and Immunology, Institute of Biology, University of Campinas. All experimental procedures were conducted in male or female mice from 8−12 weeks of age that were housed in regular filter-top cages with *ad libitum* access to sterile water and food and a maximum of 5 mice per cage. The experimental protocols were approved by the Ethics Committee on Animal Use (CEUA) at the Institute of Biology, University of Campinas (protocol 5882-1/2021, 6129-1/20–22). The mice received via gavage 200  μL of a mixture of the following antibiotics at the indicated concentrations: 5 mg/mL neomycin (Sigma Aldrich #N6386), 5 mg/mL gentamicin (Sigma Aldrich, #G3632), 5 mg/mL ampicillin (Sigma Aldrich #A0166), 5 mg/mL metronidazole (Sigma Aldrich #M3761), and 2.5 mg/mL vancomycin (Sigma Aldrich #V2002). Antibiotics were administered at 8 a.m. daily for 3 or 7 d as indicated. The control mice were gavaged with 200 μL of PBS. For the AIMD experiments, animal facility chow (Nuvilab #CR-1) containing 20% insoluble fiber and 0.2% soluble fiber was used. For the inulin diet experiments, control or 10% inulin diets were purchased from Research Diets Inc. (#D10012-M and #D19071901, respectively) or Rhoster indústria e comércio LTDA, Brazil.

GF mice were generated on a C57BL6/J background in the Disease Modeling Unit at the University of East Anglia, UK. All experimental procedures were conducted in male or female mice from 8 to 12 weeks of age that were held in isolators in the germ-free facility. All experiments were approved by the Animal Welfare and Ethical Review Body (AWERB, University of East Anglia, Norwich, UK) and were performed following the guidelines of the National Academy of Sciences (National Institutes of Health publication 86-23, revised 1985) and the provisions of the Animals (Scientific Procedures) Act 1986 (ASPA) under UK Home Office approval (PP9417531). GF mice received tributyrin (Sigma Aldrich #W222322) by oral gavage at a concentration of 3 g/kg or glycerol (Sigma Aldrich #G5516) for 7 d. Additionally, a group of GF mice received antibiotic treatment (same mixture and period as previously described: 200 μL containing 5 mg/mL neomycin, 5 mg/mL gentamicin, 5 mg/mL ampicillin, 5 mg/mL metronidazole, and 2.5 mg/mL vancomycin, administered daily at 8 a.m. for 3 d). Another group of GF mice received fecal matter from SPF mice or from human donors classified as aged or young by gavage for colonization. For this purpose, the young donors were 18−35 y old, and the old donors were > 65 y (see human samples below). Fecal samples (~ 100 mg) were homogenized in 2 mL of degassed sterile PBS and filtered through a 70 µm filter. The male mice were gavaged with 200 µL of this fecal matter once and sacrificed 3 weeks later. The mice were euthanized, and samples were collected three weeks after fecal matter transplantation. Additionally, a group of young (8−12 weeks) and aged mice (1.5 y old) was included in the experiments under SPF conditions at the Quadram Institute.

For the mono-colonization experiments, male C57BL6/J background germ-free mice (Taconic #B6NTac-GF) were bred in-house and moved into a positive pressure cage system at 3 weeks old. After this, the mice were colonized with *Bacteroides ovatus* (ATCC #8483) or *Bacteroides thetaiotaomicron* (ATCC #29148) by gavage inoculation with 1 × 10^9^ CFU/mL in a volume of 100 µL. Bacterial feces colonization was confirmed by homogenizing feces in 1 mL of sterile PBS, diluting the samples serially and then plating them on plates of supplemented brain heart infusion medium (BHIS). These mice were then fed with a control diet or a 10% inulin diet for three weeks (Research Diets Inc. #D10012-M and #D19071901, respectively).

C57BL/6J wild type was obtained from Janvier and GPR109A KO (originally described as PUMA-G KO) mice were obtained from Prof. Stefan Offermans.^25^^,^^26^ These mice were bred and housed at UPS44-TAAM (CNRS, Orleans, France) in a temperature-controlled animal facility. The mice were anesthetized using isoflurane or CO_2_ and euthanized by cervical dislocation prior to collection of samples.

### Human samples

This research was performed in accordance with the Declaration of Helsinki, the standards of the International Conference on Harmonization (ICH) Good Clinical Practice (GCP), and national law. The collection, storage, and use of all human tissue samples were carried out within the terms of the Human Tissue Act 2004 (Human Tissue Authority). All human samples for this research were accessed via the Norwich Research Park Biorepository (Human Tissue Authority-Licensed Tissue Bank, License number 11208) under the ethics approval granted by the National Health Service Health Research Authority (NHS HRA) (East of England-Cambridge East Research Ethics Committee, REC Ref 24/EE/0119; IRAS number 340288).

Fecal samples from young individuals were obtained from donors that provided full written informed consent under Biorepository Access Committee project numbers (GUMMY study Reference BAC.011. A4). Fecal samples from aged individuals were obtained from the MOTION study (IRAS 241617) that was reviewed and provided a favorable opinion from HRA East Midlands – Nottingham 1 Research Ethics Committee (19/EM/0055) and reviewed and approved by the Quadram Institute Bioscience Human Research Governance Committee, Ref QIB04-2018. The study's clinical trial registration number is NCT04199195 registered at https://clinicaltrials.gov/. Participant recruitment took place from October 2019 to February 2023. All participants provided written informed consent to participate. The fecal samples were chosen by random from these repositories.

Colonic tissue samples were obtained from the Norfolk and Norwich University Hospital Foundation Trust diagnostic archive without specific consent from participants (the FOCUS study Reference BAC.017.24) in line with the generic HRA approval of the NRP Biorepository on the use of diagnostic archives by researchers.

### Histology and immunofluorescence

#### Human samples

Sections from formalin-fixed paraffin-embedded (FFPE) samples were used for immunofluorescence with primary antibodies against MUC2 (Santa Cruz Biotechnology #sc-7314, 1:50) and CA1 (Santa Cruz Biotechnology #sc-393490, 1:200). After antigen retrieval in sodium citrate buffer, the slides were incubated with primary antibody overnight at 4 °C, followed by secondary antibody incubation (FITC and Cy3, Jackson, #111095-144-JIR, #111-165-144-JIR, 1:500) for 1 h at room temperature. Nuclei were counterstained with DAPI (Vector Labs, #H-1200-10).

#### Mouse samples

The proximal and distal portions of colon were collected and fixed in 4% formalin, embedded in paraffin and sectioned for staining. After sodium citrate antigen retrieval, primary antibodies against CHGA (ThermoFisher, #MA5-42925), CA1 (Santa Cruz Biotechnology #sc-393490), FABP2 (R&D Systems #AF1486), CLCA1 (Abcam #ab180851), LY6G (BioLegend #108408), MCT1 (Santa Cruz Biotechnology #sc365501), MCT4 (Santa Cruz Biotechnology #sc-376465), OATP (Santa Cruz Biotechnology #sc-271157), or KI67 (Abcam # ab15580) were diluted in antibody diluent (Dako #S0809) at 1:200 dilution for most antibodies (1:2000 for KI67), and the tissue sections were incubated with the antibody dilutions overnight. The samples were then incubated for 1 h at room temperature with FITC-, Cy3- or Cy5-conjugated secondary antibodies (Jackson, #111-095-144-JIR, #111-165-144-JIR, #115-095146-JIR, #115-165-146-JIR, #705-165-147-JIR) diluted in antibody diluent at 1:1000 for anti-rabbit and 1:500 for anti-mouse or anti-goat. The slides were mounted with Vectashield H-1000 supplemented with DAPI (Vector Labs, #H-1200-10). To confirm the specificity of the staining, controls were processed without primary antibodies. Images were captured using Olympus BX51 microscope with IMAGEpro software or a Zeiss AxioImager M2 with ZenBlue Software.

### Crypt isolation and cell dissociation

For crypt isolation, entire colons were harvested, opened longitudinally and washed in cold PBS to remove feces. The colon was cut into smaller pieces, the pieces were divided equally into two microtubes containing HBSS-1% penicillin/streptomycin (P/S), and the tubes were kept on ice. Once all the samples were collected, EDTA was quickly added to each tube to a concentration of 10 mM, and the tubes were incubated for 20 min at 37 °C in a thermomixer at 1000 rpm. A quick shake by hand was performed every 5 min. Colon pieces were removed, and the tubes were centrifuged for 5 min at 300 × g and 4 °C. The resulting pellet was washed with cold HBSS supplemented with 0.04% bovine serum albumin (BSA) and 1% P/S to remove EDTA. After centrifugation for 5 min at 300 × g and 4 °C, the isolated pellet was resuspended in HBSS-0.04% BSA-1% P/S. To remove mucus, 10 mM dithiothreitol (DTT) was added, and the cells were incubated at room temperature for 10 min with agitation. Cold PBS-0.04% BSA was then added, the resulting suspension was then passed through a 70 µm cell strainer, and the cells were centrifuged for 5 min at 300 × g and 4 °C. The supernatant was carefully aspirated, the pellet was resuspended in TrypLE Express (Thermo Fisher) at 37 °C, and DNase at 50 µg/mL was added. The pellet was gently pipetted up and down to dissociate the cells and the suspension was incubated at 37 °C for 5 min. After dissociation, the reaction was stopped by adding HBSS-0.04% BSA-1% P/S, followed by the addition of cold PBS-0.04% BSA. The suspension was then filtered through a 40 µm cell strainer, and the tubes were centrifuged for 5 min at 300 × g. The supernatant was aspirated, and the pellet was resuspended in cold PBS-0.04% BSA. This protocol was used for the isolation of IECs for subsequent analyses by single-cell RNA sequencing.

### Droplet-based single cell gene expression

The single-cell suspensions were loaded promptly onto a Chromium Next GEM Chip G (10X Genomics, #CG000388) following the manufacturer's protocol, aiming to capture ~8,000 cells per sample. Briefly, GEM generation was performed in the Chromium Controller, followed by post-GEM clean-up and cDNA amplification. We carried out all 3’ gene expression library construction (#PN-1000196). Quality control of the libraries was carried out using the high-sensitivity chip of the Bioanalyzer. The libraries were sequenced in paired-end mode using an Illumina HiSeq, with a read length of 150 bp, targeting a sequencing depth of ~20,000 mean reads per cell. Quality control metrics including the actual number of recovered cells, median genes per cell, and mean reads per cell, are provided in Supplementary Table 1.

### Analysis of scRNA-seq data

The FASTQ files were processed using *Cell Ranger v6.0.1* (10X Genomics), which aligned the reads to the *mm10* (version 2020A) mouse transcriptome (10X Genomics) and extracted the cell barcodes and unique molecular identifiers (UMIs). Gene expression matrices were generated with a count pipeline with standard arguments and filtered to exclude data from poor-quality cells, as outlined below. First, we estimated cell-free mRNA contamination and removed it with the *SoupX* library^27^ by comparing the raw and filtered matrices obtained with Cell Ranger. Next, we excluded genes that were expressed in fewer than 3 cells and cells with fewer than 200 detected genes using Seurat v.4.1.0.^28^ We used the *scDblFinder* library^29^ to remove doublets and excluded cells with a percentage of mitochondrial genes greater than 25% (**Supplementary Table 1**). To correct for variability arising from technical and biological effects, we used the *IntegrateData*^30^ function in *Seurat* using the sample ID as co-variable.

After the dimensionality reduction of integrated data performed by *RunPCA* followed by *RunUMAP*, the cell-type identification was performed by graph clustering (Louvain). We used the *FindAllMarkers* function in Seurat to determine the marker genes for each cluster. Using the Wilcoxon Rank Sum test and adjusted *p*-values calculated based on Bonferroni correction, we identified clusters containing highly discriminative marker genes that were previously identified in mouse or mouse orthologues of those identified in humans (Supplementary Tables 2 and 3). We performed annotations by manual analysis of the main marker genes in the clusters as described above.

To identify epithelial cells, we initially examined markers that differentiated them from immune and stromal cells, as described in Supplementary Table 3. We then classified the epithelial cells into absorptive, secretory, and proliferative subtypes using established markers.^31^ We subsequently assigned the cells to specific subtypes, including enterocytes, immature enterocytes, goblet cells, cycling transient amplifying (cycling TA) or stem cells, as well as less common cell types, such as tuft cells and enteroendocrine cells. To achieve this goal, we used a combination of known markers from the literature^32^^,^^33^ and databases such as *PanglaoDB* and *CellMarker*^34^^,^^35^ (Supplementary Table 3). Finally, we excluded clusters that lacked markers for any cell type of interest or contained low-quality cells. The absorptive secretory cells were defined by subsetting the absorptive subtypes (enterocytes and immature enterocytes) with normalized expression of *Muc2* > 3.

Publicly available scRNA-seq datasets were obtained from the gene expression omnibus (GEO) repository (Supplementary Table 1). These datasets were utilized for subsequent analysis to explore cell type-specific gene expression patterns. For the Nyström et al. dataset,^36^ the focus was specifically on the cell types characterized by them, utilizing the same marker genes identified in their study. To integrate this dataset with our own, we used the Seurat function *IntegrateData*, with sample IDs included as a covariate to account for batch effects. For the Correa et al.^22^ and GutCellAtlas dataset,^37^ the analysis was conducted directly on the downloaded data as provided, without any further modifications.

The *Slingshot*^38^ library was utilized to perform a pseudotime analysis on the integrated data. The analysis focused on absorptive lineage. The scripts for downstream pseudotime analysis are described below.^39^

To estimate the relative differences in cell-type proportions between different experimental conditions, we utilized *Speckle v0.0.3*^40^ software.

For specific cell-type differential expression analysis, we employed^28^ the default Seurat differential gene expression testing framework with a corrected *p*-value cut-off *p* < 0.05. To infer the biological processes in which the genes are involved, we utilized the *ClusterProfile* library^41^ for gene ontology analysis of genes that had an average log2-fold threshold of 0.25 and an adjusted *p*-value less than 0.05. The gene ratio was defined as the proportion of genes associated with that term relative to the total number of significant genes in the analysis. Supplementary Table 4 lists DEGs.

We used the *AddModuleScore*^42^ function in Seurat to calculate the absorption and glycosylation scores. We utilized a list of defined transporters for each nutrient^43^ (Supplementary Table 3). The glycosylation score was calculated using a predefined list of glycosylation-related genes (Supplementary Table 3).

### Tissue staining with lectin probes

Slides were incubated in a mixture of Ulex europaeus agglutinin I (UEA-1 fluorescein conjugated, Vector Labs # FL-1061-5) at a 1:1000 dilution and wheat germ agglutinin 1 (WGA1, Alexa Fluor 633 conjugated, Thermo Fisher #W21404) at a 1:500 dilution in lectin wash buffer (LWB; 50 mM Tris-HCl (pH 7.4), 150 mM NaCl, 2 mM MgCl_2_, 1 mM CaCl_2_) for 2 h in the dark. The slides were mounted using Vectashield H-1000 supplemented with DAPI (Vector Labs). Staining was performed using UEA1 alone, UEA1 combined with WGA1, or UEA1 following immunofluorescence staining, depending on the experimental setup. When combined with immunofluorescence, the UEA1 staining protocol was applied after completion of the immunofluorescence procedure. Quantitative analysis of the stained sections was performed with ImageJ software.

### Quantification of inter-crypt absorptive goblet cells

Quantification of UEA1-positive cells was performed to assess their distribution within the inter-crypt region. Hybrid absorptive-secretory cells expressing CA1 and stained with UEA1 (*bona fide* inter-crypt absorptive goblet cells) were consistently observed in the inter-crypt space across multiple images (>10). Based on these observations, we proceeded with the quantification of UEA1⁺ cells, specifically within the inter-crypt region.

To match the normalization strategy used in the single-cell analysis, we initially quantified CA1⁺UEA1⁺ cells as a fraction of total CA1⁺ absorptive cells, which revealed a 2.4-fold increase upon AIMD. To validate the use of UEA1 staining for broader quantification, we then normalized the number of inter-crypt UEA1⁺ cells per crypt and observed a comparable fold change. The similarity between these independent measurements supports the use of inter-crypt UEA1⁺ cell counts as a reliable proxy for assessing the number of inter-crypt absorptive goblet cells.

For each sample, at least four sections were analyzed. Counting was performed in a blinded manner, without knowledge of cohort information, to ensure unbiased results. Only inter-crypt UEA1-positive cells with an enterocyte-like morphology were included in the analysis. The cell counts were normalized to the number of crypts. Quantitative analysis of the stained sections was conducted using ImageJ (v.68).

### Colonic whole-mounted tissue

Proximal portions of the colon from the mice were affixed flat onto filter paper, and the mucus layer was removed by vigorously flushing the epithelial surface. The tissues were then fixed in 4% neutral buffered formalin for 3 h, permeabilized with 0.1% Triton X100 for 15 min and washed with PBS. The tissues were subsequently stained to visualize mucus secretion using UEA-1 fluorescein-conjugated stain at a 1:500 dilution for 30 min. The cell membranes were stained with CellMask Deep Red plasma membrane stain (Thermo Fisher Scientific) at a 1:1000 dilution for 10 min at room temperature, and the nuclei were stained with DAPI. All the staining procedures were performed under agitation. Images were captured using a Molecular Devices ImageXpress Micro Confocal system and processed with Imaris image analysis software. Inter-crypt absorptive goblet cells were manually identified using the Spots function in Imaris. Five images from each sample were analyzed, and the averages of these measurements were used for group comparisons.

### Butyrate measurements from fecal samples

Briefly, approximately 15 mg of fecal samples were homogenized for 1 min at 6000 RPM using a Precellys® 24 Touch homogenizer (Bertin Technologies) in 300  μL of 0.1% orthophosphoric acid (Lichropur) diluted in deionized water. The samples were centrifuged at 15,000 RPM for at least 20 min or until the samples were clarified. Internal standards were added, and the samples were transferred to high-performance liquid chromatography (HPLC) vials (Chromex Scientific). Butyrate content was then quantified by LC–MS/MS using an Agilent 6490 Triple Quad MS mass spectrometer (Agilent Technologies, Santa Clara, CA, USA) equipped with an Agilent 1290 HPLC system (Agilent Technologies, Santa Clara, CA, USA) using the described method.^44^

### 16S rRNA gene sequencing and analysis

Bacterial DNA was extracted from the colonic luminal content using the PureLink™ Microbiome DNA Purification Kit (Thermo Fisher, #A29789). 16S gene sequencing targeting the V3–V4 region was performed using Illumina technology. Data were processed using the *nf-core/ampliseq* pipeline with the SILVA 138 database, including quality filtering, read merging, and taxonomic assignment. The taxa present in less than 20% of the samples were filtered out. Alpha diversity and beta diversity (PCoA using Bray–Curtis distance) were calculated using the *phyloseq* and *microbiome* packages in R. Differential abundance analysis was performed using LEfSe with the *microbiomeMarker* package.

### Statistical analysis

All the statistical analyses were conducted in R (Version 4.1.2) and the statistical significance was determined at *p* < 0.05. Prior to analysis, the data were checked for normality, and parametric tests (student *t*-tests and ANOVA) or non-parametric tests (Wilcoxon and Kruskal‒Wallis tests) were used for group comparisons. Box plots show the mean (center), 25th–75th percentiles (box), and 5th–95th percentiles (whiskers); outliers are displayed individually. For bar plots, error bars represent the standard error of the mean (SEM). All raw count values from microscopy analyses and butyrate measurement are provided in Supplementary Table 5.

## Results

### Acute antibiotic-induced microbiota depletion affects the transcriptional profile of intestinal epithelial cells at a single-cell level

**Figure 1.**
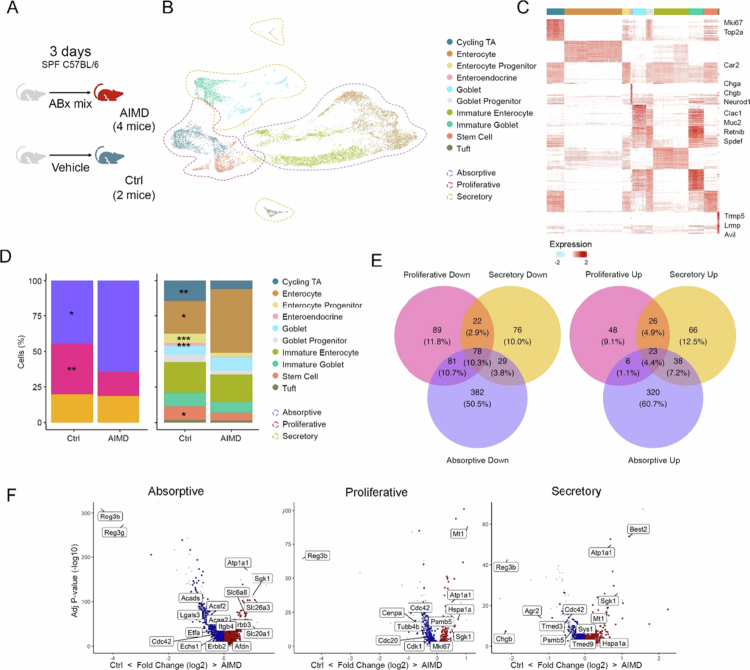
Antibiotic-mediated microbiota depletion (AIMD) reshapes the colon epithelium. (A) Schematic representation of the experimental design illustrating the treatments performed in the mice. (B) UMAP plot of scRNA-seq data from colonic IEC, combined data sets from AIMD and control. The dashed lines delineate the proliferative (red), absorptive (lilac), and secretory subgroups (orange) of intestinal epithelial cells (IECs), while colors are used to distinguish the various epithelial cell types. (C) Heatmap from B of the top 50 markers for each cell type. Marker genes were identified using Wilcoxon statistical test with significance threshold adjusted *p* < 0.05. (D) Proportion of the defined populations for broad epithelial classification in left and cell type in right, in control and AIMD groups. Statistics were calculated by Speckle. * Adjusted *p* < 0.05. ** Adjusted *p* < 0.01. *** Adjusted *p* < 0.001. (E) Venn diagram depicts the overlapping DEGs between the absorptive, proliferative, and secretory subsets. Left: downregulated genes; Right: upregulated genes. Significance was determined by MAST statistical test with a significance threshold adjusted *p* < 0.05 and log2-fold change > 0.25. (F) Volcano plots highlight specific DEGs from E in the indicated populations. Red and blue indicate DEGs that are specifically upregulated or downregulated in each population, respectively, while shared DEGs are shown in gray.

To investigate the cell-type specific response of the colonic epithelium to acute microbiota depletion, we treated mice with an antibiotic cocktail over a 3-d course (Figure 1A) and used single-cell mRNA-seq (scRNA-seq) to profile cells from control and antibiotic-induced microbiota-depleted (AIMD) mice (*n* = 2 controls, *n* = 4 AIMD). AIMD mice showed a substantial decrease in luminal bacterial DNA, reduced microbiota diversity, and altered composition, consistent with previous studies^45^^,^^46^ (Supplementary Figure 1). We analyzed 16,217 colonic epithelial cells (5,129 controls and 11,058 AIMD) (Figure 1B, Supplementary Figure 2A, Supplementary Table 1), identifying distinct cell types: proliferative (stem cells, cycling TA and progenitors), secretory (goblet cells, immature goblet cells, enteroendocrine cells, and tuft cells), and absorptive (enterocytes and immature enterocytes) cells with specific transcriptional profiles (Figure 1B, C, Supplementary Table 2).

We investigated the effects of AIMD treatment on the proportions of different colonic epithelial cell types and their gene expression profiles. Our analysis revealed significant alterations in the proportions of absorptive, proliferative, and secretory epithelial cell subsets between the AIMD and control groups (Figure 1D). Notably, AIMD mice presented a decrease in proliferative cells, comprising less than half of the proportion observed in control mice, accompanied by an increase in absorptive cells. However, no significant differences were observed in the proportion of secretory cells. Additionally, there was a significant reduction in the number of enteroendocrine cells, cycling TA, stem cells, and enterocyte progenitor cells, as well as a significant increase in mature enterocytes following antibiotic treatment (Figure 1D).

We explored the gene expression changes induced by AIMD across proliferative, absorptive, and secretory cell subsets. While several differentially expressed genes (DEGs) were shared across all subsets, such as those encoding AMPs (e.g., *Reg3b* and *Reg3c*) and membrane transporters (Figure 1E and F, Supplementary Figure 2B, Supplementary Table 4), most gene expression changes were cell subset specific. Proliferative cells presented downregulation of genes associated with cell cycle progression and mitotic metaphase (e.g., *Cdc20*, *Cenpa*, *Cdk1*, and *Tubb4b*), whereas absorptive cells showed downregulation of genes related to the citric acid cycle, lipid metabolism pathways related to SCFA metabolism (e.g., *Acaa2, Acsf2, Acadl, Acads*), and upregulation of genes involved in intestinal epithelium organization (e.g., *Cdh1*). Secretory cells displayed decreased expression of genes involved in vesicle transport (e.g., *Tmed3, Tmed9*) and mucus production and secretion, including downregulation of *Muc2* (Supplementary Figure 2B and C, Supplementary Table 4).

Consistent with the scRNA-seq data (Figure 1D), there was marked suppression of proliferating cells, as indicated by Ki67 staining (Supplementary Figure 2D), and of the number of enteroendocrine cells following antibiotic treatment (Supplementary Figure 2E). Notably, there was also a decrease in the expression of genes coding for enzymes involved in serotonin and incretin production in the enteroendocrine population (Supplementary Figure 2F).

Next, we investigated whether the gene expression changes observed after AIMD were due to the direct action of the drugs or the loss of microbiota. To address this, we generated an scRNA-seq dataset from the colonic IECs of germ-free (GF) mice (*n* = 2 mice; 7,449 cells) and compared it with the scRNA-seq data of SPF (specific pathogen-free) mice (Supplementary Figure 2G–I, Supplementary Table 1). In proliferative cells, we observed a reduction in cell cycle progression (Supplementary Figure 2I, Supplementary Table 4), and in secretory cells, there was a reduction in gene expression related to vesicle transport and mucus secretion, similar to what we detected in AIMD (Supplementary Figure 2I, Supplementary Table 4). Absorptive cells presented increased gene expression related to energy metabolism and absorption (Supplementary Figure 2I, Supplementary Table 4). There was an overlap of DEGs across all cell subsets of both the GF and AIMD groups compared with the control group, indicating that many of the observed changes were related to the microbiota and not due to the direct effect of antibiotics on the intestinal epithelium (Supplementary Figure 2H and I). Notably, the greatest number of gene expression changes both in AIMD mice compared with untreated control mice and between GF and specific pathogen-free (SPF) mice were observed in absorptive cells (Figure 1E, Supplementary Figure 2H).

### Enterocyte functional specialisation depends on microbiota

**Figure 2.**
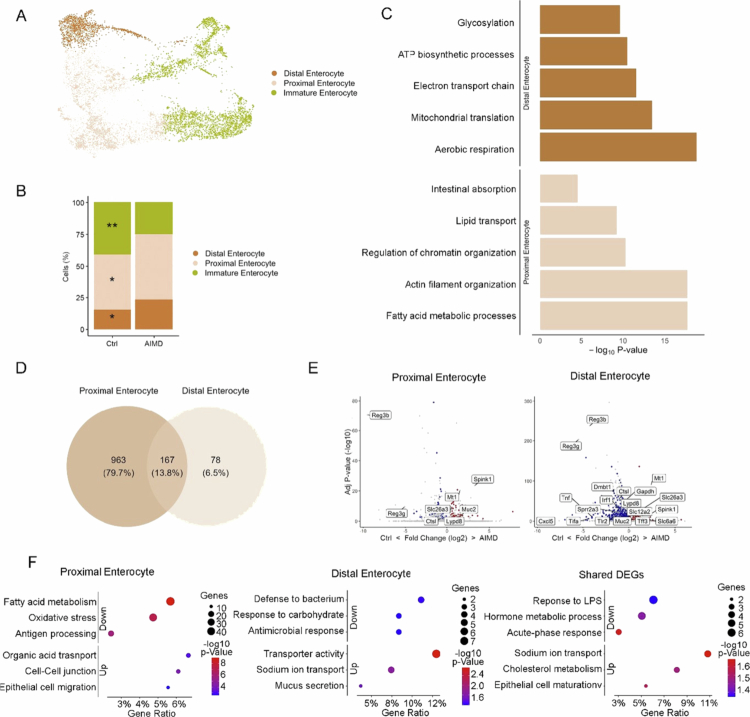
The maturation and function of absorptive cells are modulated by microbiota depletion. (A) UMAP plot of the scRNA-seq data from the absorptive population, combined AIMD and control data sets. The colors indicate the specific cell types within the absorptive population. (B) Proportion of the indicated cell types within the absorptive population. Statistics were calculated by Speckle. * Adjusted *p* < 0.05. ** Adjusted *p* < 0.01. *** Adjusted *p* < 0.001. (C) GO analysis of marker genes for the indicated cell types within the absorptive population. Marker genes were identified using Wilcoxon statistical test with a significance threshold adjusted *p*-value < 0.05 and log2-fold change > 0.25. (D) Venn diagram shows the overlapping DEGs among the cell types within the absorptive population. The Wilcoxon statistical test with a significance threshold of adjusted *p* < 0.05 and log2-fold change > 0.25 was used. (E) Volcano plots highlight specific DEGs from D in each cell-type of the absorptive population. Red and blue indicate DEGs that are specifically upregulated or downregulated, respectively, while shared DEGs are shown in gray. (F) Dot plot of GO enrichment analysis for DEGs specific to cell types within the absorptive population shown in E, displaying adjusted *p*-values and GeneRatio.

Since we found a predominance of gene expression modulations in the absorptive population upon AIMD and in germ-free mice (Figure 1E, Supplementary Figure 2H), we explored these cells in further detail in AIMD mice. We identified three different subsets of absorptive cells, immature enterocytes, proximal colon enterocytes, and distal colon enterocytes, based on literature-defined genes (Figure 2A, Supplementary Table 3).

There was a reduction in the immature population (Figure 2B), indicating that the maturation of absorptive cells is sensitive to microbiota depletion. Proximal colon enterocytes presented increased expression of genes involved in metabolism and transport, suggesting a key role in absorption (Figure 2C). In contrast, distal colon enterocytes were more strongly associated with energy metabolism, indicating functional specialization associated with enterocyte localization (Figure 2C).

**Figure 3.**
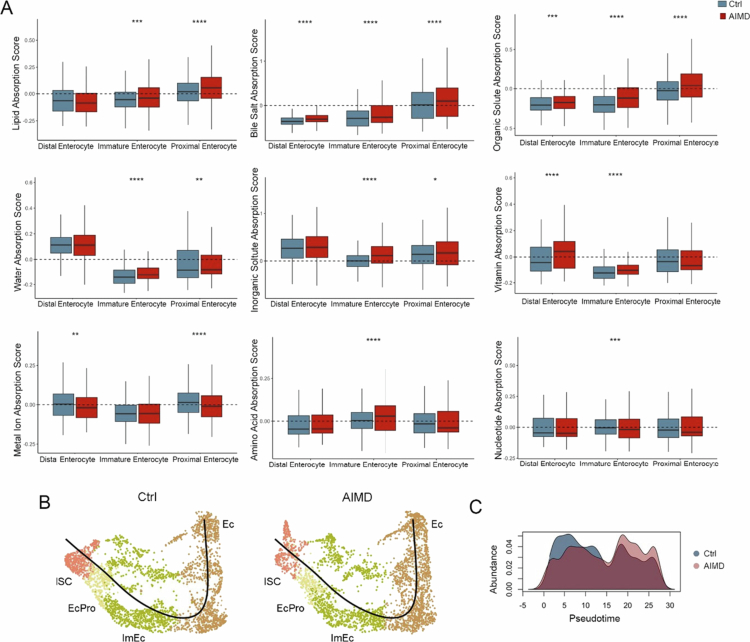
Increased expression of absorptive genes after antibiotic treatment. (A) Absorption scores as indicated on the Y-axis for various nutrients across cell types in the AIMD (red) and control (gray) groups; statistical significance was determined by Wilcoxon test; ****p < 0.0001, ***p < 0.001, **p < 0.01, *p < 0.05. For the box plots, the center represents the mean, the box represents the 25th–75th percentile, and the whiskers are 5th–95th percentile. (B) Pseudotime analysis of the absorptive population. UMAPs were split by condition (AIMD vs Ctrl) and normalized to the same number of cells. ISC: intestinal stem cells; EcPro: enterocyte progenitor; ImEc: immature enterocyte; Ec: enterocyte. (C) Distribution of cells within the absorptive lineage in control and AIMD mice across pseudotime. Kolmogorov‒Smirnov test, *p* < 0.05.

Specific gene expression changes were observed upon antibiotic treatment for proximal and distal enterocytes, with a considerable number of DEGs shared between both populations (Figure 2D and E, Supplementary Table 4). Proximal colon enterocytes were the most affected by AIMD (Figure 2D and E, Supplementary Table 4), suggesting that they are more sensitive to microbiota depletion. Gene ontology analysis revealed that proximal colon enterocytes presented a reduction in the expression of genes related to antigen presentation and lipid metabolism, whereas increased expression of genes related to the maintenance of the intestinal barrier and transport through the membrane and digestive system was detected (Figure 2F). Distal colon enterocytes showed downregulation of genes involved in response to microbes and carbohydrate metabolism, and upregulation in genes associated with the transport of sodium (Figure 2F). The DEGs shared between the proximal and distal enterocyte populations upon AIMD indicated a shift in their expression profiles, transitioning from an external stimulus‒response pattern to a more pronounced metabolic and absorptive profile. Indeed, a significant increase in the gene expression of several nutrient transporters was detected in enterocytes upon AIMD (Figure 2E and F). To quantify these changes, we applied a gene expression-based score using absorption-associated genes to assess the degree of functional adaptation in enterocytes following microbiota depletion (Figure 3A).

Using this approach, we detected the upregulation of genes encoding transporters for lipids (e.g., *Fabp2, Acsl3, Abcg2, Plin2, Apob*), bile salts (*Slc10a2, Slc51b, Slc51a*), organic and inorganic solutes (*Slc13a2, Slc44a1, Slc26a2*), and water (*Aqp8, Slc5a1*) in AIMD mice (Supplementary Table 4). The observed increase in transporters, particularly in immature and proximal enterocytes, suggests a compensatory mechanism to maintain absorption under altered conditions, highlighting the dynamic plasticity of enterocytes in response to microbiota depletion.

To further explore the impact of the microbiota on colonic epithelium turnover, we analyzed the differentiation trajectory of enterocytes from intestinal stem cells to mature intestinal epithelial cells in control and AIMD mice (Figure 3B). Cells from AIMD mice tended toward later pseudotime values (Figure 3C). This observation is consistent with compromised cell turnover in AIMD, indicating alterations in epithelial homeostasis in the absence of a microbiota.

### Microbiota-dependent regulation of absorptive inter-crypt goblet cell population

Notably, among the genes shared between the proximal and distal enterocyte populations upon AIMD, the genes associated with mucus formation and secretion, including *Muc2*, *Agr2* and *Clca1* (Figure 2E), which are typically markers of goblet cells, were upregulated. Our scRNA-seq analysis revealed a marked increase in the *Muc2*^+^ population in the absorptive cell population upon AIMD compared with controls (Supplementary Figure 3A).

We next focused on performing an in-depth characterization of these *Muc2*-positive cells found in the cluster of absorptive cells (Supplementary Figure 3B and C). These cells exhibit a unique gene expression profile, expressing not only marker genes of both immature and mature absorptive cells (such as *Fabp2, Ly6g, Car1*, and *Mxd1*) but also markers of mucus-secreting goblet cells (*Muc2, Agr2, Clca1*) (Supplementary Figure 3B and C, Supplementary Table 2). A pseudotime-based development trajectory analysis indicated that these secretory features in absorptive cells arise early during differentiation (Supplementary Figure 3D and E). Using the absorption score, we found that *Muc2*^*+*^-absorptive cells presented absorptive potential, albeit lower than that of canonical enterocytes but significantly greater than that of canonical secretory cells (Supplementary Figure 4A). These cells presented elevated expression of genes associated with lipid uptake, organic solute transport, and bile salt absorption, while *Muc2*^*+*^-absorptive distal cells were more involved in water absorption (Supplementary Figure 4B). In a recent study, Nyström et al. identified intercrypt goblet cells as cells expressing both absorptive markers and mucus components.^36^ These cells generate intercrypt mucus differing from the crypt plume mucus secreted by canonical goblet cells, which is more penetrable to smaller molecules. We integrated our scRNA-seq dataset with the dataset published by Nyström et al. (1,755 and 4,614 cells from two distal colon samples enriched for *Muc2*^+^ cells) and identified the cell populations previously described in their study (Supplementary Figure 5A).

Integration confirmed robust alignment between datasets (Supplementary Figure 5B). Canonical goblet cells overlapped with the secretory population in our dataset, whereas both noncanonical goblet cell subsets clustered within the absorptive lineage (Supplementary Figure 5C). Consistently, our *Muc2*^+^ absorptive cells followed the same pattern, overlapping with both non-canonical subsets: *Mxd1*^–^ cells aligning with immature enterocytes and *Mxd1*^+^ cells aligning with mature enterocytes (Supplementary Figure 5D, E). Because the increase in the number of these cells appeared to be consistent with a general enhancement of absorptive function in the colon upon AIMD and given the notable inferred expansion of these cells upon AIMD, we further explored these recently recognized but poorly characterized cells and their links to the microbiota.

Inter-crypt goblet cells have been shown to secrete mucus that is stained with lectin Ulex Europaeus agglutinin 1 (UEA1, binding fucose), but not with wheat germ agglutinin 1 (WGA1, binding to *N*-acetylglucosamine/sialic acid).^36^ Indeed, WGA1 labeled only canonical goblet cell mucus produced inside the crypt, whereas the UEA1 lectin effectively detected mature mucus-producing cells in the upper crypt and luminal regions (Supplementary Figure 6A). We confirmed the expression of a specific set of glycosyltransferases in the *Muc2*^+^ absorptive cells from our data sets (Supplementary Figure 6B and C, Supplementary Table 3), which expressed genes predominantly involved in fucosylation (e.g., *Fut2*) and O-glycosylation (*Galnt3*, *Galnt4*, *C1galt1*, *C1galt1c1*), but no consistent upregulation of sialyltransferases (*St3gal6*, *St6galnac2*, *St6galnac3*, *St6galnac4*, *St6galnac6*) was detected (Supplementary Figure 6B and C). This pattern suggests a distinctive glycosylation profile that may underlie the UEA1 reactivity of these cells.

Using antibodies against key proteins linked to absorption and markers of goblet cells, including the mucus lectin UEA1, we detected double-positive cells at the top of the crypts and between crypts in the proximal region (Supplementary Figure 7A–C, note that some of these markers stain different compartments within the cell, so no overlap of signals is expected). Additionally, using LY6G, a marker of distal colon enterocytes, and CLCA1, we identified co-stained cells in the distal colon, demonstrating that their presence is not restricted to the proximal colon (Supplementary Figure 7D).

In summary, our scRNA-seq data revealed an increase in *Muc2*^+^ absorptive cells upon AIMD, which we conclude represent intercrypt goblet cells and exhibit a hybrid absorptive-secretory profile. This finding seems consistent with the overall change in gene expression towards a more absorptive nature of the colon epithelium upon AIMD.

**Figure 4.**
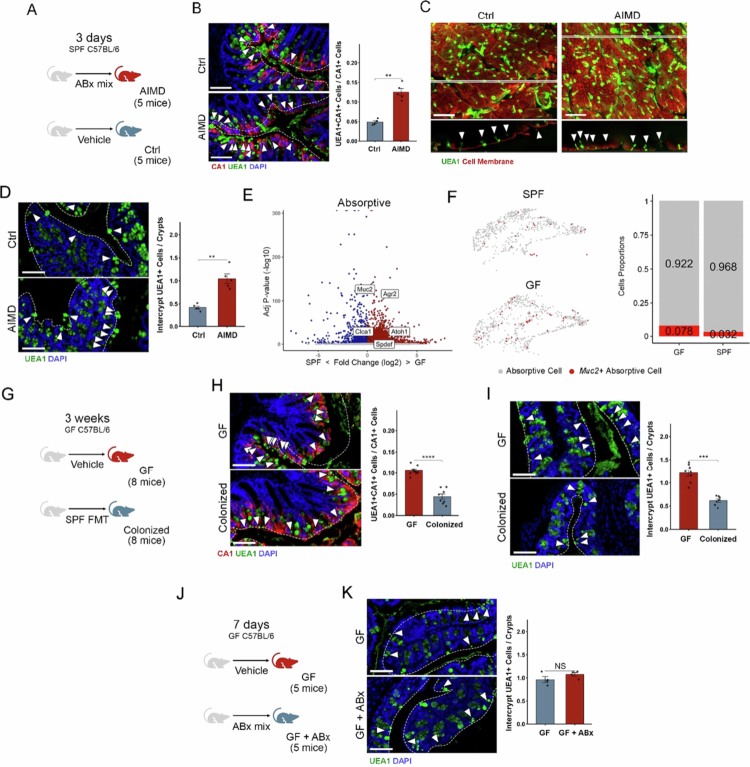
Absorptive inter-crypt goblet cells are intricately linked to the microbiota. (A) Schematic representation of the experimental design illustrating the AIMD mix treatment performed in SPF mice. (B) Immunofluorescence staining of proximal colonic sections from control and AIMD mice using anti-CA1 and UEA1 lectin for mucus and DAPI for DNA counterstaining. Quantification of CA1^+^UEA1^+^ cells within total CA1^+^ cells. Scale bar, 50 µm; *n* = 5 per group. (C) Whole-mount proximal colon from control and AIMD mice stained with the UEA1 lectin for mucus and CellMask for the cell membrane. Representative Z-stacks from the region outlined by the white box are shown at the bottom. UEA1^+^ cells in inter-crypt region are indicated with arrows. Scale bars, 20 µm. (D) Staining of proximal colonic sections from control and AIMD mice with the UEA1 lectin for mucus and DAPI for DNA counterstaining. UEA1^+^ cells in the inter-crypt region are indicated with white arrows and the epithelial surface is indicated by the white dashed line. Quantitation of these cells is shown on the right. Scale bar, 50  µm; *n* = 5 in each group. (E) Volcano plot showing DEGs in the absorptive populations from GF and SPF mice. Some goblet cell markers are highlighted in the figure. The Wilcoxon statistical test with a significance threshold of adjusted *p* < 0.05 and log2-fold change > 0.25 was used. (F) UMAP plot of the scRNA-seq data showing the *Muc2*^+^ population (red dots) within the absorptive cluster in both the GF and SPF mice, grey dots represent canonical absorptive cells (enterocytes and immature enterocytes). The right panel shows the proportion of *bona fide* absorptive inter-crypt goblet cells. (G) Schematic representation of the experiment with FMT from SPF to GF mice. (H) Immunofluorescence staining of proximal colonic sections from SPF colonized and GF mice with anti-CA1, UEA1 lectin for mucus, and DAPI for DNA counterstaining. UEA1^+^ cells in the intercrypt region are indicated with arrows, and the epithelial surface is indicated by a white dashed line. Quantification of CA1^+^UEA1^+^ cells within total CA1^+^ cells. Scale bar, 50 µm; *n* = 8 per group. (I) Staining of proximal colonic sections from SPF colonized and GF mice with UEA1 lectin for mucus and DAPI for DNA counterstaining. UEA1^+^ cells in inter-crypt region are indicated with arrows, and the epithelial surface is indicated by the white dashed line. Quantitation of UEA1^+^ cells per crypt is shown on the right. Scale bar, 50 µm; *n* = 8 in each group. (J) Schematic representation of the experimental design illustrating the ABx (antibiotics cocktail) treatment performed in GF mice. (K) Proximal colonic sections from GF control and GF treated with antibiotics-cocktail mice stained with UEA1 lectin for mucus and DAPI for DNA. UEA1^+^ cells in inter-crypt region are indicated with arrows, and the epithelial surface is indicated by the white dashed line. Quantitation of UEA1^+^ cells per crypt is shown on the right. Scale bar, 50 µm; *n* = 4 in each group. All error bars are SEM.

Next, we investigated the role of the microbiota in modulating inter-crypt goblet cell numbers in the colonic tissue by immunostaining proximal colon sections stained with CA1 (carbonic anhydrase, a paradigm marker for absorptive cells expressed in non-canonical inter-crypt goblet cells, Supplementary Figure 7B) and UEA1, which revealed a greater proportion of UEA1⁺CA1⁺ cells embedded within the canonical enterocyte (CA1⁺) population in AIMD mice (Figure 4A and B).

Whole-mount colon staining with UEA1 additionally highlighted an increase in the number of UEA1 stained cells in the inter-crypt region, following AIMD treatment (Figure 4C). Complementary analyses using lectin staining further supported these findings, showing a greater proportion of UEA1⁺WGA⁻ cells compared to UEA1⁺WGA⁺ canonical goblet cells (Supplementary Figure 8A). We assessed the proportion of inter-crypt UEA1⁺ cells per crypt (Figure 4D) and found that their number reliably reflects the overall expansion of the UEA1⁺CA1⁺ cell population (Figure 4B), and we used the latter more generally for quantification (see methods section for details). This expansion was also observed in antibiotic-treated female mice, indicating a robust and consistent response to microbiota depletion that is independent of sex (Supplementary Figure 8B). Interestingly, analysis of distal colon sections revealed greater numbers of these cells compared with the proximal colon, but AIMD induced only a small change in their abundance, suggesting that this phenotype is primarily localized to the proximal colon (Supplementary Figure 8C).

Although the expansion of UEA1^+^ inter-crypt cells was observed following acute AIMD (3 d), we assessed the persistence of this phenotype under extended antibiotic treatment (7 d). Remarkably, the increase in these absorptive-secretory hybrid cells was maintained after prolonged antibiotic exposure (Supplementary Figure 8D and E), highlighting the stability and robustness of this response to microbiota depletion.

Next, we used our scRNA-seq data from GF mice (see above) to confirm whether the significant expansion of inter-crypt goblet cells is reflected by the loss of microbiota. Focusing on the gene expression changes in absorptive cells, we observed an upregulation in markers of goblet cells (Figure 4E) and an increase in the population of *Muc2*^+^ cells in the absorptive cell population (Figure 4F) in GF mice, suggesting an increased number of these non-canonical absorptive goblet cells. We detected a significant decrease in UEA1⁺CA1⁺ cells within the CA1⁺ enterocyte population following SPF-microbiota colonization in both male and female GF mice compared to GF controls (Figure 4G and H, Supplementary Figure 8F). This finding confirms that the presence of microbiota suppresses inter-crypt goblet cell numbers. The overall proportion of inter-crypt UEA1⁺ cells per crypt decreased after the colonization of GF mice (Figure 4I), validating this metric as a reliable and reproducible measure of noncanonical (absorptive) inter-crypt goblet cell expansion. We next investigated whether this phenotype persists after antibiotic withdrawal. The mice maintained for three weeks following AIMD still presented an expanded inter-crypt goblet cell population (Supplementary Figure 8G and H). This persistence is consistent with incomplete recovery after antibiotic treatment and with evidence of sustained dysbiosis (Supplementary Figure 1E–G).^47^ To further identify the direct effects of the drugs used on AIMD, we treated GF mice with the antibiotics mix and did not identify any difference in the inter-crypt goblet cell numbers (Figure 4J–K).

### Microbiota-derived butyrate suppresses inter-crypt goblet cell numbers via butyrate and its receptor, GPR109A

**Figure 5.**
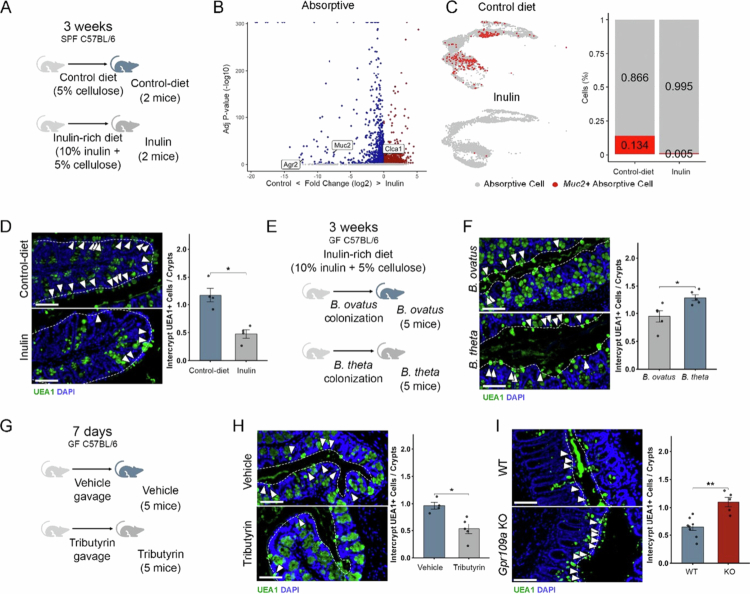
Microbiota-derived butyrate reduces absorptive inter-crypt goblet cell numbers through GPR109A. (A) Schematic representation of the experiment using the inulin-rich and control diet treatments performed in SPF mice. (B) Volcano plots highlighting DEGs within the absorptive cell populations of inulin-rich and control diet-fed mice. Some goblet cell markers are highlighted in the figure. The Wilcoxon statistical test with a significance threshold of adjusted *p* < 0.05 and log2-fold change > 0.25 was used. (C) UMAP plot of the scRNA-seq data highlighting the *Muc2*^*+*^ cell population within the absorptive cluster in both inulin-rich and control diet-fed mice. The gray dots represent canonical absorptive cells (enterocytes and immature enterocytes), while red dots represent *Muc2*^*+*^ cells. The right panel shows the proportion of *Muc2*^*+*^ cells. (D) Staining of proximal colon sections from inulin-rich and control diet-fed mice with the UEA1 lectin for mucus and DAPI for DNA counterstaining. UEA1^+^ cells in inter-crypt region are indicated with arrows, and the epithelial surface is indicated by a white dashed line. Scale bar 50 µm, *n* = 4 in each group. (E) Schematic representation of the experimental design illustrating the inulin-rich diet administration and mono-colonization experiments performed in GF mice. (F) Staining of proximal colon sections from *B. ovatus* or *B. thetaiotaomicron ('B. theta')* mono-colonized mice fed an inulin-rich diet with UEA1 lectin for mucus and DAPI for DNA counterstaining. UEA1^+^ cells in inter-crypt region are indicated with arrows, and the epithelial surface is indicated by a white dashed line. Scale bar, 50 µm; *n* = 5 in each group. (G) Schematic representation of the experiment using tributyrin supplementation of GF mice. (H) Staining of proximal colon sections from vehicle or tributyrin supplemented GF mice with UEA1 lectin for mucus and DAPI for DNA counterstaining. UEA1^+^ cells in inter-crypt region are indicated with arrows, and the epithelial surface is indicated by a white dashed line. Scale bar 50 µm; *n* = 4/5 in each group. (I) Staining of proximal colon sections from *Gpr109a* WT and KO mice with the UEA1 lectin for mucus and DAPI for DNA counterstaining. UEA1^+^ cells in inter-crypt region are indicated with arrows, and the epithelial surface is indicated by a white dashed line. Scale bar 50 µm, *n* = 8/6 in each group. All error bars are SEM.

Because the microbiota generates a plethora of metabolites that, in turn, affect the host, such as short-chain fatty acids, by fermenting plant fibers, we ventured to identify pathways affecting inter-crypt goblet cells. Our previous work demonstrated that an inulin-rich diet modulates the intestinal epithelium anatomy in a microbiota-dependent manner.^22^ This diet significantly altered the bacterial composition in the colon and promoted SCFA production, whereas the control diet for these experiments contained only 5% cellulose, resulting in a marked reduction in luminal SCFA levels.^22^ Analysis of scRNA-seq data from 15,432 colonic IECs of mice fed an inulin-rich or control diet revealed a decrease in the expression of genes associated with mucus secretion within the absorptive population compared to mice on the control-diet (Figure 5A and B, Supplementary Table 4) (*n* = 2 inulin-rich diet-fed mice, 2 control-diet fed mice). Notably, the number of absorptive *Muc2*^*+*^ cells was significantly lower in mice subjected to the inulin-rich diet relative to control-diet-fed animals (Figure 5C). This reduction in *bona fide* inter-crypt goblet cells was further confirmed by UEA1 staining, which revealed a marked decrease in UEA1^+^ inter-crypt cells in inulin-fed mice compared with those in control diet-fed mice (Figure 5D).

Because an inulin-rich diet increases SCFA-producing microbes,^48^ we asked whether metabolites derived from inulin bacterial fermentation regulate the number of inter-crypt goblet cells. To test this hypothesis, we mono-colonized mice with two distinct *Bacteroides* species that ferment fibers differently and fed them with an inulin-rich diet. We observed that mice monocolonized with *Bacteroides ovatus*, a species associated with butyrate production,^49^^,^^50^ exhibited a reduced number of inter-crypt goblet cells compared to mice mono-colonized with *Bacteroides thetaiotaomicron*, a species not typically linked to butyrate production^51^ when both were subjected to an inulin-rich diet (Figure 5E and F). Given the metabolic differences between these bacteria, particularly with respect to butyrate production, we investigated whether butyrate is specifically involved in the regulation of inter-crypt goblet cell numbers. We treated GF mice with tributyrin, a butyric acid precursor established to increase the butyrate concentration in the colon.^52^ We observed that tributyrin-fed GF mice presented a decrease in the proportion of inter-crypt goblet cells (Figure 5G and H), indicating that this metabolite is sufficient to induce the suppression of these absorptive-secretory hybrid cells.

Butyrate affects intestinal physiology by providing an energy source, acting as a histone deacetylase inhibitor and binding to G-protein coupled receptor (GPCR) proteins, activating several cellular pathways.^53^ We investigated the role of GPR109A, a receptor known to be involved in butyrate-signaling. UEA1 staining in the colons of GPR109A knockout (KO) mice revealed an increase in inter-crypt goblet cells compared to wild-type control mice (Figure 5I). These findings suggest that butyrate acts as a regulator of inter-crypt goblet cell numbers through the activation of GPR109A. We also tested the potential involvement of GPR43, which is more efficiently activated by acetate and propionate than by butyrate, using a knockout model for this receptor. The absence of GPR43 did not affect inter-crypt goblet cell numbers under homeostatic conditions (Supplementary Figure 9A), suggesting that this receptor is not involved in their regulation. This finding reinforces that butyrate specifically via GPR109A rather than SCFAs in general, plays a key role in the microbiota-driven regulation of these cells.

In addition to regulating absorptive-secretory cell numbers, butyrate-GPR109A influences epithelial cell proliferation. Ki67 staining revealed that GPR109A knockout mice exhibited reduced proliferation (Supplementary Figure 9B), while tributyrin supplementation increased proliferative activity (Supplementary Figure 9C). This finding suggests that butyrate, through GPR109A, modulates epithelial renewal, potentially balancing non-canonical inter-crypt goblet cell number suppression with increased enterocyte differentiation to maintain tissue homeostasis.

### Age promotes expansion of inter-crypt goblet cell population of human and mouse colon epithelium

**Figure 6.**
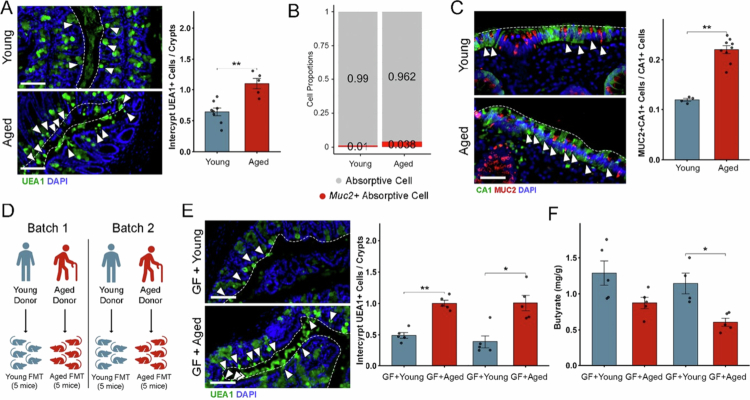
Absorptive inter-crypt goblet cell numbers are associated with aging (A) Staining of proximal colon sections from aged (~80 weeks old) and young (8–12 weeks old) mice with UEA1 lectin for mucus and DAPI for DNA counterstaining. UEA1^+^ cells in the inter-crypt region are indicated with arrows, and the epithelial surface is indicated by a white dashed line. Scale bar 50 µm, *n* = 8/5 in each group. (B) Proportion of *Muc2*^+^ cells in the absorptive cluster in aged (>65 y, *n* = 3) and young-adult (<65 y, *n* = 4) human colonic samples. Analysis was performed using the Gut Cell Atlas dataset. (C) Immunofluorescence staining of young and aged human descending colonic sections using anti-CA1 and anti-MUC2 antibodies and DAPI for DNA counterstaining. Double-stained cells in inter-crypt region are indicated with arrows, and the epithelial surface is indicated by a white dashed line (please note that MUC2 and CA1 are expressed in different compartments within a cell). Quantification of CA1^+^MUC2^+^ cells within total CA1^+^ cells on the right. Scale bar, 30 µm; *n* = 5/8 per group. (D) Schematic representation of the experimental design for human fecal microbiota transplantation (FMT) in germ-free (GF) mice. The donors were all healthy, female (41 and 72 y, batch 1) and male (35 and 77 y, batch 2). (E) Staining of proximal colon sections from GF mice colonized with human microbiota from aged or young donors with the UEA1 lectin for mucus and DAPI for DNA counterstaining. Two independent experiments were performed using FMT from different donors for each group, as illustrated in D. UEA1^+^ cells in inter-crypt region are indicated with arrows, and the epithelial surface is indicated by a white dashed line. Scale bar, 50 µm; *n* = 5 in each group. (F) Quantification of butyrate levels in the colonic luminal content of GF mice colonized with human microbiota from aged or young donors, as described in D. Statistical significance was determined by unpaired *t*-test; ****p < 0.0001, ***p < 0.001, **p < 0.01, *p < 0.05. *n* = 5 per group. All error bars are SEM.

Aging is commonly associated with changes in the microbiome, including reduced microbial diversity and decreased production of metabolites such as butyrate, in mice and humans.^54^^,^^55^ Therefore, we tested whether old age is linked to changes in inter-crypt goblet cell numbers. Indeed, we found that aged mice (1.5 y) also exhibit a significantly greater number of inter-crypt goblet cells compared to young adult mice (8−12 weeks) do (Figure 6A).

Given the conservation of inter-crypt goblet cells in humans, we took advantage of publicly available data sets encompassing scRNA-seq data from the human colon epithelium across different ages to investigate whether its abundance is also influenced by aging in humans.^37^ We identified an inter-crypt goblet cell-like absorptive secretory population in the single-cell transcriptome data from the adult human colon with an expression profile pattern similar to that observed in mice, with markers of both goblet cells and enterocytes (Supplementary Figure 10A–E). We confirmed the presence of inter-crypt goblet cells in human colonic tissue sections, using co-staining for CA1 and MUC2, showing the expression of mucin by enterocytes in the inter-crypt region (Supplementary Figure 10F). Remarkably, we noted age-related differences in the scRNA-seq dataset: aged donors (more than 65 y) had a greater presence of the absorptive *Muc2*^+^ cell population (Figure 6B). We confirmed this observation histologically by co-staining MUC2 with CA1 in colonic tissue sections of aged and young donors (Figure 6C).

To further investigate the role of the microbiota in modulating this phenotype, we performed fecal microbiota transplantation (FMT) from two healthy elderly donors (>70 y) and two adult donors (approximately 40 y) into germ-free mice, with five germ-free mice per group (Figure 6D and E, Supplementary Figure 11A, Supplementary Table 5). The results were consistent across batches: mice receiving FMT from the elderly donors presented a significantly greater number of UEA1^+^ intercrypt cells and lower butyrate concentrations compared to those receiving FMT from young adults (Figure 6E and F).

These findings suggest that the increase in inter-crypt goblet cell numbers in aged individuals is driven by microbiota composition and resulting changes in butyrate rather than aging alone. The reduced SCFA-producing capacity of the aged microbiota likely contributes to this effect, further supporting the role of butyrate in regulating these absorptive-secretory hybrid cells.

## Discussion

This study demonstrated how gut microbiota manipulations affect the microanatomy of the colon epithelium and the transcriptional profile of various IEC. Our scRNA-seq analysis following acute microbiota depletion and in germ-free mice revealed both shared and cell-type specific gene expression responses. This, in turn, highlights cellular diversity even within a particular cell type, such as absorptive enterocytes, and the key role of the microbiota in shaping gene expression and cellular plasticity in the colon epithelium. Upon AIMD, we detected changes in cell proportions, as validated by histochemistry, such as a significant decrease in proliferating cells, enteroendocrine cells and an increase in numbers of inter-crypt goblet cells and an increase in the proportion of mature absorptive cells. With respect to enteroendocrine cells, we found that AIMD caused not only a decrease in their number but also a notable change in their expression of a number of key genes encoding incretins, hormone-like peptides that are involved in regulating aspects such as peristaltics and appetite. These findings are in line with the recognized role of enteroendocrine cells in bridging the microbiota and colon physiology.^56^

Recent studies have highlighted not only anatomical and functional differences between intestinal segments, such as the small intestine, cecum, and colon, but also regional specialization within each compartment.^57–59^ Although our single-cell dataset encompasses the entire colon, we were able to distinguish proximal and distal epithelial populations, particularly within the absorptive lineage, using distinct marker combinations.^58^ We used the single cell transcriptomic data to infer spatial specialization in the response to the microbiota, with proximal colon enterocytes showing a distinct response to microbiota depletion compared to distal enterocytes. Overall, we observed a change from an external stimulus-response pattern to a more metabolic and absorptive profile in the enterocyte population, suggestion a pronounced functional shift. Remarkably, upon microbiota-depletion, we observed the expression of mucus-related genes within the absorptive cell population, suggesting that microbial depletion reveals a latent secretory program in these cells. In line with this, we identify *Muc2* expressing cells in the absorptive population and relate these cells with previously described non-canonical inter-crypt goblet cells,^36^ characterized by the expression of enterocyte-associated genes and a specific fucose-rich mucus. Here, we expand on this idea by identifying and characterizing this population of cells along the colonic epithelium, from proximal to distal regions. Pseudotime analyses revealed that secretory features arise early during the differentiation of these cells. Consistently, the population we defined as *Muc2*⁺ absorptive cells overlaps with both mature and immature compartments. These cells express key genes involved in mucus production and secretion (e.g., *Muc2*, *Agr2*, *Clca1*) alongside with high levels of absorption-related genes (e.g., *Fabp2*, *Car1*, *Slc16a1, Slc16a3*, *Slc26a3*). Histological analysis confirmed the co-localization of mucus lectins and absorption-related proteins within the same cells, which were located in the inter-crypt regions along the colonic epithelium.

Previously, inter-crypt goblet cells were described as constituents of the normal colonic epithelium.^36^ In our study, we observed a marked increase in the number of these inter-crypt goblet cells under abnormal microbial states, such as acute AIMD and in GF mice. Conversely, a fiber-rich diet in mice with an intact microbiota, or monocolonized with *Bacteroides ovatus*, a bacterium capable of metabolizing fiber into butyrate, or the supplementation of germ-free mice with tributyrin, a pro-drug of butyrate, markedly reduced the number of these cells.

Although initially considered rare, we describe that absorptive inter-crypt goblet cells represent approximately 5% of absorptive cells, as measured by immunostaining, highlighting their physiological relevance even under steady-state conditions. Our findings suggest that the abundance of these cells is tightly regulated by microbially derived butyrate, which acts through the G-protein-coupled receptor GPR109A, as demonstrated in mice deficient in this receptor. This molecular axis is well known for its role in modulating inflammation and preventing cancer in the colonic epithelium.^60^^,^^61^ Hence, our data suggest that these absorptive-secretory cells may be functionally involved in disease processes and should be further explored in the context of pathological epithelial remodeling. In fact, a recent study suggested the upregulation of inter-crypt goblet cells upon infection with the pathogenic bacterium *Streptococcus gallolyticus.*^62^

Consistent with regional specialization, nutrient absorption predominates in the proximal colon (although it remains significantly less absorptive than in the small intestine), and water absorption occurs mainly in the distal colon, the inter-crypt goblet cells follow a similar distribution pattern, showing distinct marker profiles in the proximal versus distal regions. Notably, their abundance and regulation appear to be linked to butyrate availability, which is greater in the proximal colon.^63^^,^^64^ In contrast, the distal colon, where butyrate levels are lower, harbors a greater proportion of these cells. This spatial regulation became particularly evident in antibiotic-treated mice, where the impact of butyrate depletion was most pronounced in the proximal colon. These findings suggest that regional differences in microbial metabolites contribute to shaping epithelial plasticity in a compartment-specific manner, supporting our focus on mechanistic exploration in the proximal colon. Notably, Nyström and colleagues analyzed mainly the distal colon, where inter-crypt goblet cells are enriched and less responsive to antibiotic-induced microbiota depletion.^36^ As shown by our functional experiments, butyrate/GPR109A signalling negatively regulates the differentiation of these cells. This regional difference helps reconcile our findings with those of previous reports. We did not further investigate hybrid cell features in the cecum or small intestine, although these regions may be of interest for future studies.

Our data demonstrate that the expansion of inter-crypt goblet cells is reversible within relatively short time frames and tightly linked to microbial signals. GF mice reverted the phenotype after 3 weeks of FMT from SPF donors; tributyrin supplementation for 7 d significantly reduced their abundance; and inulin supplementation for three weeks similarly decreased their levels compared to controls. By contrast, three weeks after antibiotic withdrawal recovery of normal inter-crypt absorptive goblet cell numbers remained incomplete, consistent with more persistent changes to the microbiome composition after antibiotics treatment affecting microbial function, as has been published previously.^47^ Together, these findings position inter-crypt goblet cells as context-dependent players in epithelial adaptation, with potential implications for gut barrier integrity and inflammatory disease susceptibility. The precise function of these cells, whether protective, pathological, or both, remains to be determined, as ways and tools are needed to specifically delete these cells, which need yet to be developed.

The gut microbiome undergoes significant changes over the lifespan, with early postnatal shifts followed by stabilization in adulthood and a progressive loss of microbial diversity in aged individuals.^65^ This microbial decline has been linked to increased frailty and impaired gut barrier function in elderly individuals (reviewed in).^66^

We found that the number of inter-crypt goblet cells increased in aged mice and in human colonic samples from older individuals. Our results support a model in which age-associated microbiota alterations,^55^ particularly reduced SCFA production, promote the expansion of absorptive inter-crypt goblet cells. It is tempting to speculate that this may reflect an adaptive or maladaptive response to barrier dysfunction during aging, potentially increasing susceptibility to inflammatory conditions such as colitis and other age-related diseases.^55^^,^^67^ The infant gut microbiome is also strongly susceptible to perturbation by antibiotics treatment with potentially long-lasting effects, e.g., in terms of metabolic health, in a mouse model and humans.^68–71^ Therefore, future studies should illuminate the dynamics of absorptive inter-crypt goblet cells upon antibiotics-mediated microbiota perturbation in infants in a mouse model and in humans.

In conclusion, our results illustrate the plasticity of the colon epithelium in adapting to the microbial environment. We focused on a newly identified absorptive secretory cell type that is regulated by the presence and composition of the microbiota and identified a microbially generated metabolite, butyrate, and its sensor, GPR109A, as key regulators. This might represent only a small slice of the regulatory toolbox that connects colon epithelium plasticity to the microbiota. However, our findings highlight the important role of butyrate not only as an energy source in this tissue but also as a signaling molecule that shapes colon epithelium anatomy.

### Limitations of this study

Our human microbiota transfer experiments in mice showed that the microbiota impacts on inter-crypt goblet cell numbers, and these experiments complement those in which we counted these cells from older and younger donors and demonstrated changes in their numbers during aging. As there is a stochastic element in microbiota changes during aging,^55^ a large number of donors should be analyzed in the future together with their microbiomes to elucidate the precise microbial changes that drive these alterations in cell numbers. While we have identified GPR109A as a critical element in regulating inter-crypt goblet cells, future experiments need to address the precise downstream pathways by which GPR109A promotes inter-crypt goblet cell numbers.

## Supplementary Material

Supplementary material Supplementary information

## Data Availability

All data needed to evaluate the conclusions in the paper are present in the paper and/or the Supplementary Materials. Sequence data generated in this study have been uploaded to the NCBI repository BioProject: PRJNA1136851, 16S amplicon sequencing of Antibiotic-Induced Microbiome-Depleted and Specific Pathogen Free Mice; BioProject: PRJNA1136828, Single-Cell Gene Expression Profiling of Intestinal Epithelial Cells in Colon Tissue from Antibiotic-Induced Microbiome-Depleted, Specific Pathogen Free, and Germ-Free Mice
